# Variation in Soil Fungal Composition Associated with the Invasion of *Stellera chamaejasme* L. in Qinghai–Tibet Plateau Grassland

**DOI:** 10.3390/microorganisms7120587

**Published:** 2019-11-20

**Authors:** Wei He, Andrew Detheridge, Yongmei Liu, Lei Wang, Haochen Wei, Gareth W. Griffith, John Scullion, Yahui Wei

**Affiliations:** 1 Key Laboratory of Resource Biology and Biotechnology in Western China, Ministry of Education, College of Life Sciences, Northwest University, Xi’an 710069, China; weiyahui@nwu.edu.cn; 2 School of Chemical Engineering, Northwest University, Xian 710069, China; 3 Institute of Biological, Environmental and Rural Sciences, Aberystwyth University, Aberystwyth SY23 3FL, UK; andrew.detheridge1@virgin.net (A.D.); gwg@aber.ac.uk (G.W.G.); 4 College of Urban and Environmental Science, Northwest University, Xi’an 710069, China; liuym@nwu.edu.cn (Y.L.); montez@nwu.edu.cn (L.W.); 5 Research School of Biology, Australian National University, Canberra, ACT 2601, Australia

**Keywords:** *Stellera chamaejasme* L., soil fungal community, soil physiochemical parameters, invasion, amplicon sequencing

## Abstract

*Stellera chamaejasme* L. is the most problematic weed in China’s grasslands. Its root exudates affect co-occurring plants and thus may also affect soil fungi. Soils (0–20 cm depth) on two adjacent sites, one invaded the other uninvaded, were compared for a range of physiochemical parameters and by DNA sequencing of fungal communities. At the invaded site, relationships between *S. chamaejasme* abundance, soil physiochemical factors, and fungal communities were further investigated to determine whether these relationships corroborated conclusions on the basis of site differences that could be translated into functional variation. Results showed that the invaded soils had lower N, P, organic matter, fungal alpha diversity, and relative abundance of arbuscular mycorrhizal fungi (AMF), but greater abundance of pathogenic fungi. Organic matter and P were the edaphic factors most strongly linked to site differences in total fungal communities. Within the invaded site, organic matter rather than *S. chamaejasme* cover was closely linked to total fungal composition. However, on this site, a number of fungal species that had various ecological functions and that differentiated the two sites were related to *S. chamaejasme* cover. This study indicates that lower fertility soils may be more susceptible to invasion by *S. chamaejasme*. Although the influence of *S. chamaejasme* on total fungal community composition was limited, there was evidence of effects on particular fungal species. Further research is needed to determine whether these effects influence *S. chamaejasme* invasiveness.

## 1. Introduction

*Stellera chamaejasme* L. (Thymelaeaceae) is a native perennial herbaceous plant of grasslands in China. Its remarkable acclimation ability enables it to occupy a wide range of grassland types and climates, spanning the northeast to the southwest of China [1]. In the grasslands of northern and western China, it has become an increasingly common weed [2]. It causes a deterioration of the grazing resource due to its unpalatability to herbivores and has become the most problematic weed in China’s grasslands [3], threatening the viability of pastoral farming in affected regions. Once established, it tends to persist in part due to its avoidance by grazing animals.

*S. chamaejasme* is unusual in being an indigenous invasive weed and its expansion in grasslands across China has been attributed to several factors. Poor grassland management and subsequent degradation creates opportunities for invasion [4]; for example, excessive grazing has been found to favour establishment of the species [5]. Climate warming may also favour this species [6], as it can access reserves of moisture at soil depths greater than the grasses against which it competes [7]. *S. chamaejasme* also has deeper taproots compared to other grassland species.

Allelochemicals from *S. chamaejasme* roots such as umbelliferone, mesoneochamaejasmin A, and neochamaejasmin B enhance its competitive nature and broad ecological adaptability [8]; in this regard, there is clear evidence of such chemicals inhibiting germination and/or growth of a range of grassland species [2,9,10]. Invasive plants such as *S. chamaejasme* often induce positive feedbacks from soil biotic and abiotic properties that facilitate their expansion. These interactions may contribute to changes in soil fertility. For example, Sun et al. [11] found differences in C and N cycling between *S. chamaejasme* patches and adjacent between-patch soils; patches had higher organic contents, microbial biomass and respiration, and nitrate levels. They attributed these differences to an increased amount and N content of litter produced by *S. chamaejasme*, due to reduced herbivory and deeper rooting habit improving nutrient uptake.

For exotic invasives, their uncontrolled spread is postulated to result from release constraints of soil microbial populations in their native areas and positive feedback effects on soil microbes, which further inhibit the success of indigenous members of the plant community [12]. Such effects have been demonstrated in soil sterilisation experiments [13,14,15]. In some cases, it has been possible to identify which soil microbes are affected, for example, the inhibition of ectomycorrhizal fungi in North American woodlands invaded by the exotic non-mycorrhizal herb *Alliaria petiolata* [16]. More often, however, general changes in soil microbial populations are reported, for example by the molecular analysis of fungal populations in either plant roots [17] or bulk soil [18,19,20,21]. More recent investigations have deployed DNA metabarcoding via NextGen sequencing, for example, to investigate the effect of invasive *Centaurea stoebe*, *Euphorbia esula*, and *Bromus tectorum* in prairie grasslands on soil fungal and bacterial communities [22]. DNA metabarcoding offers the potential to identify at species level which components of the microbial community are altered by weed invasion.

To date, much of the research on *S. chamaejasme* has focused on the role of allelopathic impacts on its competitive strengths (e.g., [2]). Some studies have surveyed the bacterial [23,24] and fungal [25,26] communities associated with the rhizosphere and tissues of *S. chamaejasme*. However, the focus of these studies did not extend to analyses of bulk soil and consideration of whether microbial communities may facilitate or be altered by the spread of this plant in alpine grassland habitats. Evidence that chemicals from *S. chamaejasme* have activity against phytopathogenic fungi [27] suggest at least the potential for it to directly alter soil fungal communities. Sun et al. [11] suggested that *S. chamaejasme* alters the soil nutrient status, and, further, that this is an indirect mechanism by which *S. chamaejasme* could impact fungal communities. However, it is open to question whether the invasive plants alter edaphic properties and fungal communities or vice versa, given that no controlled experiment has been performed so far. Cryptically, *S. chamaejasme* plants do not grow into the mature stage under laboratory conditions. Therefore, in this study, by employing a large-scale sampling strategy, we aimed to answer the following questions:

(1) Do soil fungal communities and edaphic properties differ between *S. chamaejasme* invaded and uninvaded sites?
(2) Does *S. chamaejasme* abundance or edaphic conditions explain variations in fungal communities?
(3) Are there differences in functional groups or particular fungal species that might relate to the presence of *S. chamaejasme*?

Here, we investigated fungal communities and key physiochemical properties in bulk soils. We compared fungal communities and associated soil properties on adjacent invaded and uninvaded grassland under similar management. Where site differences in fungal communities and soil physiochemical properties were indicated, their relationships with variations in density of cover of *S. chamaejasme* were investigated on the invaded site to establish whether site differences potentially attributable to *S. chamaejasme* were corroborated by trends within the invaded site.

## 2. Materials and Methods

### 2.1. Experimental Sites

**Figure 1:**
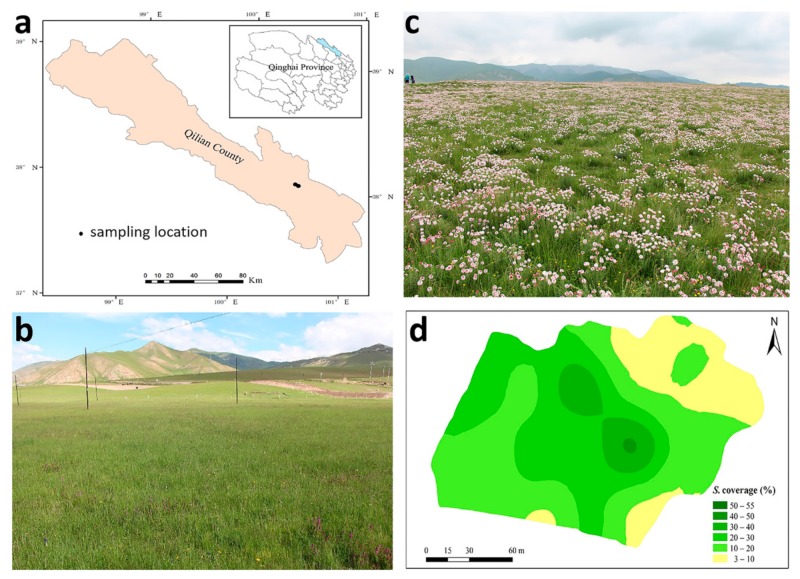
(**a**) Sampling location in Qilian County on the Qinghai–Tibetan Plateau; (**b**,**c**) uninvaded and invaded sites; and (**d**) *S. chamaejasme* coverage on the invaded site.

Two adjacent sites in Qilian County, Qinghai Province, China (38°03′9.43′N, 100°30′14.71″E) were selected for the study on the basis of their similar management, topographical characteristics, and close proximity (2.8 km) (Figure 1). Average elevation is 3169 m. Mean annual temperature is 1 °C, but in July average temperature may exceed 14 °C Annual precipitation is 420 mm, with 81% of this precipitation occurring between June and September [28]. The soil type is leptosol (FAO, Harmonized World Soil Database v 1.2) and the grassland is described as alpine meadow [29,30]. On the ‘uninvaded’ site (13,000 m^2^) *S. chamaejasme* was absent, whereas on the invaded site (23,000 m^2^) it was present at varying densities. Information provided by local farmers indicated that *S. chamaejasme* first appeared around 20 years prior to this investigation and that its coverage had slowly increased on the invaded site.

Vegetation recording and soil sampling were undertaken at 50 and 25 locations in the invaded and uninvaded sites, respectively, on the basis of systematic (approximately 20 × 20 m) grids. Between-site comparisons used 25 randomly selected data points from the invaded site. In order to increase the sensitivity of investigations into relationships between *S. chamaejasme* abundance and soil parameters on the invaded site, the full dataset was used for this aspect of the investigation. Vegetation was surveyed (total cover to 1%, and cover and number of *S. chamaejasme*) and soil samples were taken over the period of 15–17 July 2015, during the flowering season of *S. chamaejasme*. Significant rainfall occurred in the week prior to fieldwork. Plant coverage was imaged in 1 m^2^ quadrats located on a grid sampling scheme, then converted to percentage coverage of *S. chamaejasme* after normalization. As the focus of the study was on *S. chamaejasme*, the cover of other species was not recorded; for these species, only presence and general abundance was noted. Plant species common in both sites included *Agropyron cristatum* L., *Bupleurum falcatum* L., *Potentilla multifida* L., *Kobresia myosuroides* (Vill.) Fiori, *Poa calliopsis* Litv. ex Ovcz, and *Gentiana leucomelaena* Maxim. *Agropyron cristatum* L. and *Kobresia myosuroides* (Vill.) Fiori. were the dominant species on both sites. The invaded site included in addition minor occurrences of *Medicago ruthenica* (L.) Ledebour, *Oxytropis falcata* Bunge, *Echinochloa caudata* Roshev., and *Lobularia maritima* L., as well as *S. chamaejasme*. The uninvaded site vegetation also included *Taraxacum mongolicum* and *Delphinium caeruleum* [31].

### 2.2. Main Sampling and Analyses

Three soil cores (20 cm depth, 5 cm diameter) were taken at each vegetation quadrat location and bulked for soil and fungal analyses. The soil corer was thoroughly cleaned, initially with water then with 75% ethanol, between each sampling location to avoid contamination. Samples were kept at 4 °C and transferred to the laboratory within 48 h. Subsamples were either frozen at −80 °C until DNA extraction or air dried for chemical analyses.

Soil chemical analyses focused on total nutrient contents rather than more transient measures of available nutrients. Soil physiochemical properties were measured as by Bao [32], total N by the Kjeldahl method, total K by NaOH fusion-flame photometry, total P by NaOH fusion-Mo/Sb colorimetry, and soil organic matter (OM) by dichromate oxidation-FeSO_4_ titrimetry. Soil pH was measured in a 1:2.5 soil:water suspension. Gravimetric soil moisture contents were measured immediately on return to the laboratory by drying at 105 °C.

Soil DNA was extracted from 250 mg of each soil sample using an MO BIO Power Soil DNA Isolation Kit (Qiagen, Hilden, Germany) according to the manufacturer’s instructions. Polymerase chain reaction (PCR) amplification of the ITS1 (Internal Transcribed Spacer) region of the rRNA (ribosomal RNA) locus was undertaken using the primers ITS1F (5′-CTTGGTCATTTAGAGGAAGTAA-3′) and ITS2R (5′-GCTGCGTTCTTCATCGATGC-3′) linked with barcodes. The PCR reaction (25 µL) contained the following reagents: 10× PCR Buffer 2.5 µL, 10 mmol/L dNTP (deoxy-ribonucleoside triphosphate) 1 µL, forward and reverse primers (10 µmol/L) each of 1 µL, soil DNA template (50 ng/µL) 2 µL, 5 U µL^−1^ *Taq* DNA polymerase 0.4 µL, and double-distilled water (ddH_2_O) 17.1 µL. PCRs were performed at 95 °C, 5 min; 95 °C, 30 s; 50 °C, 30 s; 72 °C, 40 s (30 cycles); 72 °C, 7 min; and 4 °C hold. PCR products were purified using MoBio UltraClean PCR Clean-Up Kit (Qiagen) and an aliquot of clean PCR product for each sample was sent for sequencing. Sequencing was performed on an Illumina 2500 HiSeq (Illumina, San Diego, CA, USA) by the Beijing Biomarker Biotechnology Co., Ltd. (Beijing, China), who ligated sequencing adapters and pooled samples in equimolar concentrations for library formation.

### 2.3. Data Analysis

Differences in soil chemistry and moisture between the uninvaded and invaded sites were evaluated by ANOVA (one-way analysis of variance). Where fungal and physiochemical indices differed significantly between sites, data from within the invaded site were further investigated to assess plant–fungi–soil interrelationships.

Sequence data were processed as detailed in Detheridge et al. [33]. A total of 71 soil samples were analysed, with DNA recovered from all 25 samples on the uninvaded site and from 46 (extraction was unsuccessful in four instances) on the invaded site. Comparison between sites was on the basis of processed data from randomly selected 25 samples in the invaded group against all 25 samples from the uninvaded site. To investigate associations between *S. chamaejasme* coverage, soil properties, and fungal diversity, the analyses were performed on all the 46 invaded site samples from which DNA was extracted.

Following DNA metabarcoding of the ITS1 region between the SSU (small subunit) and 5.8S rRNA genes, the resulting sequences were assigned to taxa using the RDP (Ribosomal Database Project) Naïve Bayesian classifier [34] against the UNITE v6 database [35] supplemented with non-fungal sequences, so that non-fungi could be identified. A sequence for *S. chamaejasme* (NCBI (National Center for Biotechnology Information) accession number MG516523) was also included to identify samples where sequences belonging to this species occurred. Ecological function of fungi was assigned to each taxon (where identified) at species, genus, or family level using the Funguild database [36]. Sequence data have been submitted to the European Nucleotide Archive with reference number PRJEB29489. Shannon ($-\sum_{i=1}^{S}P_{i}lnP_{i}$ where *P_i_* = relative proportion of the *i*th taxa) and Simpson ($1/\sum_{i=1}^{S}P_{i}^{2})$ diversity indices were calculated for each quadrat.

An unconstrained ordination technique (principal coordinate analysis—PCO) was used against the Bray–Curtis distance matrix. Permutation multivariate analysis of variance (PERMANOVA) was used to determine overall significant to compare total fungal communities. Percentage abundance percentage data were transformed (square root) to reduce the effect of dominant taxa and Bray–Curtis distance matrices were calculated. The PERMANOVA used default settings with Monte Carlo *p*-values calculated on the basis of 9999 unrestricted permutations. The influence of environmental data on fungal communities was investigated by distance-based redundancy analysis (dbRDA) [37]; predictor variables were normalised before analysis. Analysis of similarity (ANOSIM) determined the extent of divergence in communities at site level and similarity percentages (SIMPER) indicated taxa contributing most to these site differences. The above analyses were performed in PRIMER 6 and PERMANOVA+ (versions 6.1.12 and 1.0.2 respectively; Primer-E, Ivybridge, United Kingdom). Analyses of variance (ANOVA) were performed in Sigma Plot (version 12.5, Systat Software Inc., San Jose, CA, USA); data normality (Shapiro–Wilk) and equality of variance (Levene’s) were tested prior to ANOVAs. Post-hoc differences were tested using Fisher’s least significant difference (LSD).

## 3. Results

Total vegetation cover was similar on the two sites (uninvaded 88.5%; invaded 87.6%), with an average of 19.8% *S. chamaejasme* cover at the invaded site (across the 50 quadrats). *S. chamaejasme* cover was highly variable, ranging from 3% to 55% with a mean of 17.5 plants/m^2^. *S. chamaejasme* was not observed in the uninvaded site (Figure 1) but sequence data showed that *S. chamaejasme* DNA was present in soils at seven quadrats (28%) from the uninvaded site compared to 33% of quadrats from the invaded site. Thus, *S. chamaejasme* was cryptically present in the uninvaded site, likely as seed or small seedlings.

### 3.1. Soil Physiochemical Properties

Uninvaded soils had higher total P, N, and organic contents (Table 1); in contrast, total K was lower in uninvaded soils. The C:N ratio of uninvaded soils was significantly higher than for soils on the invaded site. Soil pH and moisture was similar, but the latter result may have been affected by rainfall prior to sampling.

### 3.2. Fungal Populations

The mean number of sequences obtained per sample from DNA metabarcoding was 58,838 (range 17,799–279,702). After clustering, there were 3310 operational taxonomic units (OTUs) in total across all 71 quadrats where DNA was recovered.

Rarefaction was to the sample with the least number of sequences (17,800). After rarefaction, clustering (excluding those with <2 sequences) and classification, the mean fungal sequence number per sample was 16,325; non-fungal sequences (929) were not included in any further analyses.

**Figure 2:**
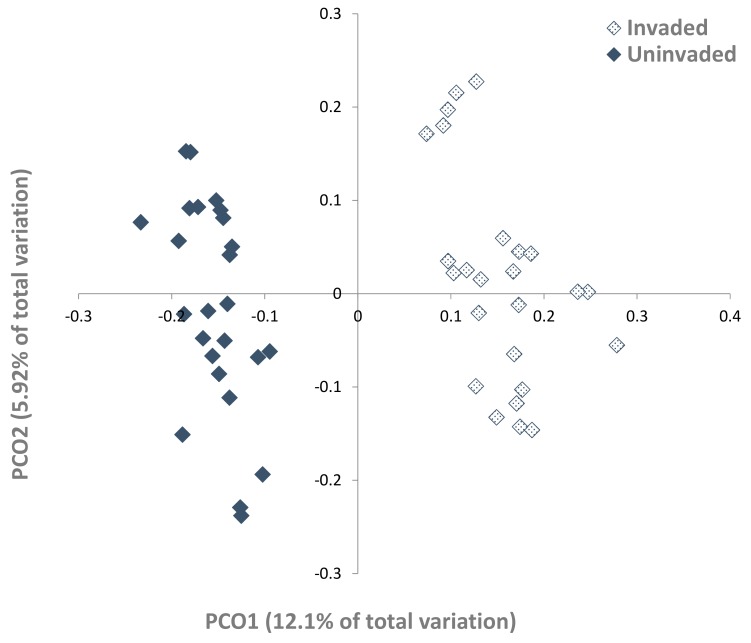
Principal coordinate ordination (PCO) of soil fungal communities in invaded and uninvaded grasslands (25 samples each site), showing a clear separation between the two communities (permutation multivariate analysis of variance (PERMANOVA) Pseudo-*F* = 7.9509, *p* = 0.001; analysis of similarity (ANOSIM) *r* = 0.509, *p* = 0.001).

The PCO plot (Figure 2) separated the fungal communities in the uninvaded and *S. chamaejasme* invaded site soils; the primary axis explained only 12.1% of the variation in these communities. The PERMANOVA main test was significant ((Pseudo-*F* = 7.9509, *p* = 0.001, *p*(MC) = 0.001), as was the ANOSIM test (*r* = 0.509, *p* = 0.001).

Both the fungal OTU count (*p* < 0.001) and the Shannon index (*p* = 0.031) were higher in the uninvaded site soils (Table 2). The relative abundances of three fungal phyla differed between sites; Glomeromycota (synonymous with AMF), Ascomycota, and Zygomycota (dominated here by species within the order Mortierellales). Ascomycota were more dominant on the invaded site than the uninvaded site (*p* < 0.001), whereas the reverse was the case for Zygomycota (*p* = 0.034) and Glomeromycota, (*p* < 0.001) which were more abundant on the uninvaded site.

**Figure 3:**
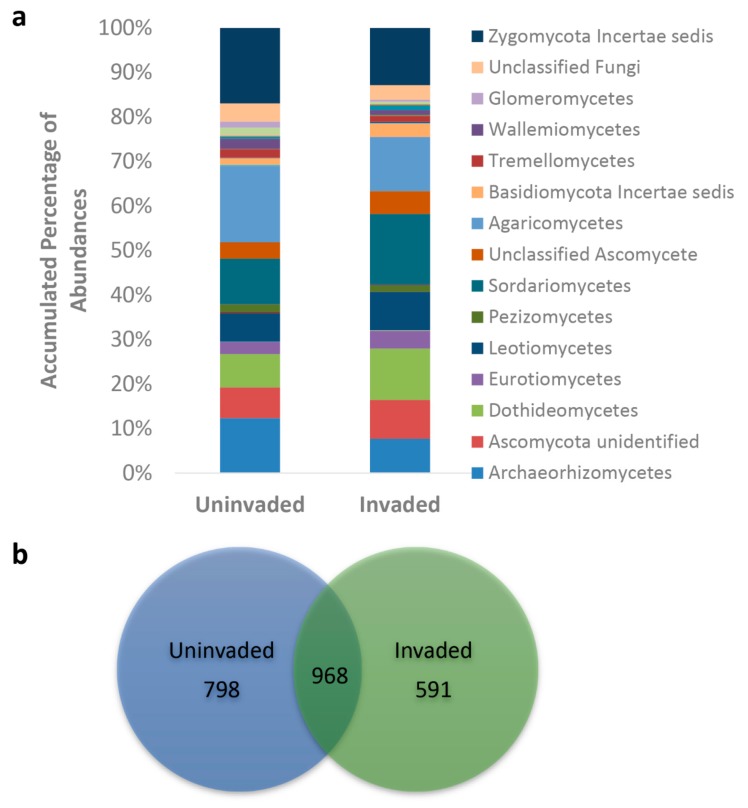
Class level differences (**a**) in mean % relative abundances and (**b**) Venn diagram of assigned OTUs (**b**) between the two (*n* = 25 each) sites.

Of those fungal classes where mean relative abundances were greater than 1%, three showed significant differences between the uninvaded and the invaded site soils on the basis of ANOVAs (Figure 3a). Leotiomycetes were higher on the invaded site (*p* = 0.01, uninvaded 6.36%, invaded 8.59%), as were Sordariomycetes (a class that includes many pathogenic species) (*p* = 0.044, uninvaded 10.2%, invaded 15.8%). On the other hand, the class Zygomycota *incertae sedis* (dominated here by the order Mortierellales) were more abundant on the uninvaded site (*p* = 0.033, uninvaded 16.9%, invaded 12.9%). The uninvaded and invaded sites had 798 and 591 distinct OTUs in each group, respectively, whereas they shared 968 OTUs in common (Figure 3b).

SIMPER analysis was undertaken to identify species and OTUs having the most influence on the separation of the communities (Table 3). An undefined *Archaeorhizomyces* species was identified as having the greatest effect on dissimilarity between the two sites. This group has been identified by Rosling et al. [38] as ubiquitous in soils and roots, although they do not seem to form recognised mycorrhizal structures. Two OTUs (OTU8 and OTU20) unidentified in the UNITE database were virtually absent from the invaded site. BLAST (Basic Local Alignment Search Tool) analysis against the NCBI database revealed that OTU8 has a high similarity to an uncultured *Mortierella* clone from arable soils (98% ID; E = 3e^−129^) and the closest named match to OTU20 is a Clavariaceae sequence from grassland soils (94% ID; E = 1e^−113^). *Humicola nigrescens* was widespread and was eightfold more abundant in the invaded site.

**Figure 4:**
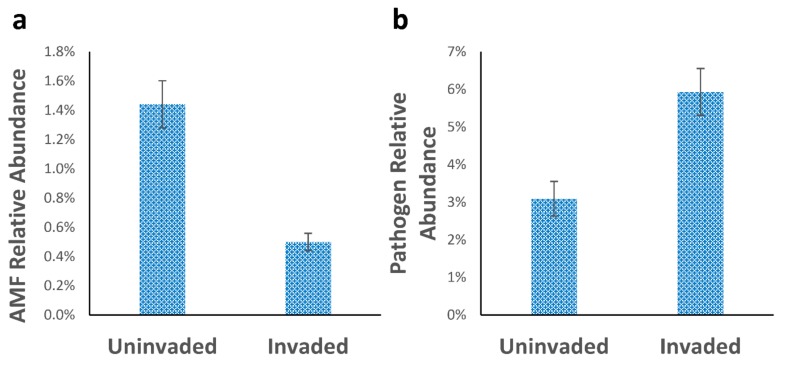
Differences between high level functional groups: (**a**) arbuscular mycorrhiza (*p* < 0.001); (**b**) pathogens (*p* = 0.003) on the basis of ANOVA (*n* = 25 per site). Error bars represent SEM (standard error of mean).

In addition to individual species, the attribution of ecological function to particular clades within the UNITE database allowed the relative abundance of functional guilds (as defined by FUNGuild) to be assessed. Arbuscular mycorrhizal fungi (phylum Glomeromycota) were more abundant on the uninvaded site (1.44% vs. 0.49%, *p* < 0.001), whereas fungi classed as phytopathogenic were more common in the invaded soils (5.93% vs. 3.09%, *p* = 0.003) (Figure 4).

### 3.3. Fungal–Soil and S. chamaejasme Interrelationships

**Figure 5:**
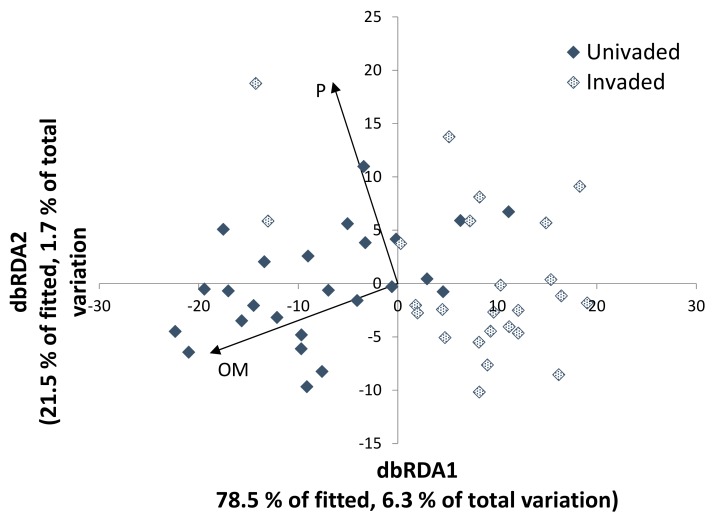
Plot relating fungal populations to soil parameters differentiating uninvaded and invaded sites. Of the parameters measured, only percentage total phosphorous and organic matter showed a significant correlation with fungal community composition (*p* < 0.01, *n* = 25 per site). dbRDA: distance-based redundancy analysis.

**Figure 6:**
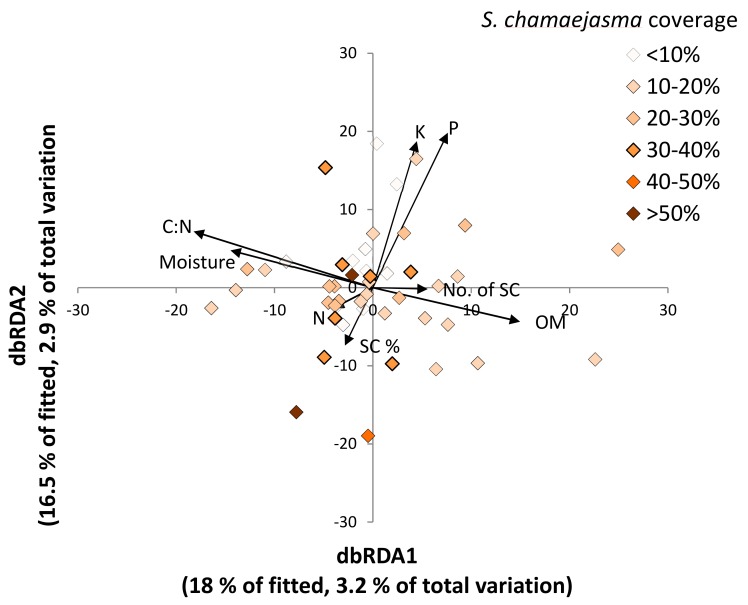
dbRDA plot of global fungal communities in the invaded site (*n* = 46) relating variations in fungal populations with soil and *S. chamaejasme* cover and abundance. Invaded site only. Nutrient, moisture and organic matter are % total (C = carbon, N = nitrogen, P = phosphorous, K = potassium, moisture, and OM = organic matter). SC = % cover and abundance of *S. chamaejasme* per quadrat; No. of SC = number of *S. chamaejasme* per quadrat.

The dbRDA analyses investigated whether soil factors were associated with differences in global fungal communities between the invaded and uninvaded sites (Figure 5). Then fungal composition was related to *S. chamaejasme* and to edaphic factors in the invaded site alone, in order to determine whether factors explaining variations within the invaded site were consistent with those differentiating sites (Figure 6). The separation of the fungal communities between the two sites was primarily along axis 1 and most closely linked to soil organic matter (OM), with higher levels of OM on the uninvaded soils (Figure 5). Within the invaded site, fungal composition varied on both axes with a wider range of soil parameters influencing this distribution; neither *S. chamaejasme* abundance nor cover were linked closely to variations in this composition (Figure 6). Indeed there was little pattern to the distribution of fungal communities; no strong link was found between measured soil parameters in these communities, and the proportion of the total variation accounted for in these analyses was very low.

**Figure 7:**
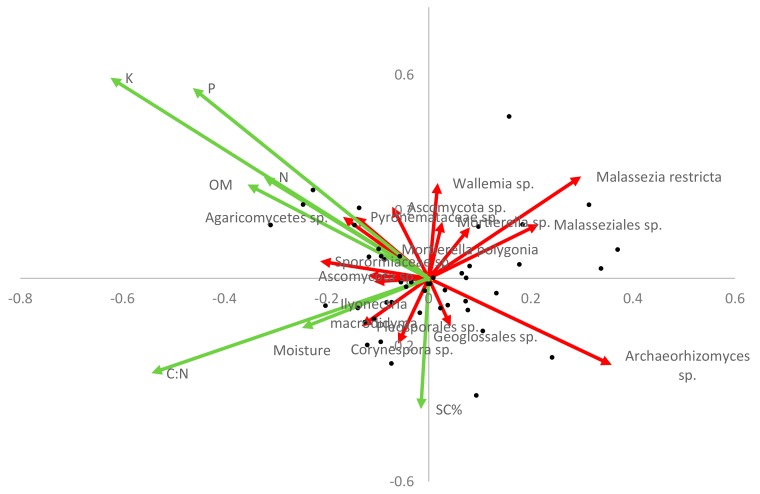
Redundancy analysis triplot of fungal species relative abundance (red) and potential predictive factors (green) for invaded site only (*n* = 46). Only those species most closely correlated with the primary axes were included. Nutrient, moisture, and organic matter are % total (N = nitrogen, P = phosphorous, K = potassium, moisture, and OM = organic matter). SC% = % cover.

Fungal community composition on the invaded site was further investigated by analysis of the relative abundance of particular species. Variation in this composition was then linked to potential drivers (edaphic and *S. chamaejasme*) (Figure 7). In some cases, there was a clear negative or positive association with nutrient fertility (e.g., *Archaeorhizomyces* sp. and Agaricomycetes sp. respectively). Several other species showed a negative correlation with *S. chamaejasme* coverage, notably *Ascomycota* sp., *Wallemia* sp. (xerophilic), and *Mortierella* sp. (saprotrophic), and a few a positive correlations, for example, *Corynespora* sp. (plant pathogen). Interestingly, several of the species identified in the SIMPER analysis (Table 3) as contributing most to the dissimilarity between sites were related to the variation in either soil parameters or *S. chamaejasme* cover (Figure 7) on the invaded site.

## 4. Discussion

Our study involved a comparison of two sites only, and thus outcomes in relation to potential influences of edaphic and *S. chamaejasme* factors on fungal populations were considered substantive only if the importance of site factors as potential drivers of fungal diversity was corroborated by evidence of similar relationships between these factors and fungi on the invaded site. The broad composition of the fungal communities, including the dominance of Ascomycota (greater on the invaded site) and Basidiomycota, and the small component as Glomeromycota were consistent with the findings of Jin et al. [26] who generated clone libraries from rhizosphere soil and root samples of *S. chamaejasme* from the Tibetan plateau.

In this paper, we found distinctive fungal communities in the grasslands where *S. chamaejasme* was present, suggesting that either the fungal community of the uninvaded site was inherently different from the invaded site prior to invasion or that the community at the invaded site had been altered by *S. chamaejasme*. Gibbons et al. [22] found the latter to be the case in an extensive investigation of the microbial communities associated with three invasive weeds of North American prairie grasslands. Here, we did not find the correlation within the invaded site between the overall fungal community and density of *S. chamaejasme*, which might have been expected if *S. chamaejasme* was driving change at this level. However, correlations between vegetation cover and overall fungal composition are often weak [39]. Nevertheless, there was some evidence that *S. chamaejasme* may affect specific rather than total fungal community indices, as some species differentiating the two sites appeared to be related to *S. chamaejasme* coverage within the invaded site, whereas others were linked more closely to variations in soil organic matter and nutrient fertility rather than *S. chamaejasme* coverage.

The site differentiation in functional groups, such as arbuscular mycorrhizal fungi (AMF) and fungal pathogens, may explain in part interactions between *S. chamaejasme* and co-occurring species. In plant communities, rare indigenous species may experience more negative feedback from pathogens compared with invasive species [40]. Invaded soils had higher relative abundance of fungal pathogens; if flavonoids in *S. chamaejasme* protect the plant from these pathogens, as suggested by Yan et al. [10], this might provide a competitive advantage over co-occurring species in soils where pathogen occurrence is high.

The relationship between plants and AMF is a complex one and may not always benefit the plant, with the potential of AMF to ‘cheat’ on their hosts [41]. In an environment where a plant species is invading native species’ habitats, AMF have been shown to facilitate both the invasion [42,43] and the ability of native plants to withstand invasion [44,45]. The impact on AMF is likely dependent on a number of factors such as soil nutrient status, host specificity, and the species of AMF present [46]. In addition to their nutritional role, AMF are also implicated in defence against plant pathogenic fungi [47,48], either by occupancy of infection loci [49] or by modification of host defence systems [50,51]. Thus, the increased pathogen presence on the invaded site may be linked at least partly to reduced AMF levels. The higher relative abundance of AMF on the uninvaded site may thus contribute to the ability of plants in this site to withstand invasion. Evidence also suggests that *S. chamaejasme* are only rarely infected with AMF [25,26], indicating that the different levels of AMF abundance may also relate to cover of this species.

Given the lower total P concentrations in invaded soils, this might have been expected to favour Glomeromycota [52], but the opposite was the case, at least in terms of relative abundance (Figure 4). *Humicola nigrescens*, which produces phytase enzymes for the mobilisation of P [53], was more abundant in the invaded site, possibly driven by the lower P reserves in these soils. In uninvaded soils, the higher abundance of Zygomycota, and particularly Mortierellales, may be linked to AMF abundance, as they mobilise inorganic P via secretion of organic acids, and act synergistically with AMF [54,55]. In addition, species from the genus *Mortierella* have recently been shown to colonise roots, promote growth, and prevent disease in *Arabidopsis* [56].

Root exudates of *S. chamaejasme* inhibit the growth of seedlings across a range of grass and broad-leaved species co-occurring on these grassland soils [2,9]. Whilst bacterial [24] and fungal [57] communities associated with the *S. chamaejasme* rhizosphere have been investigated in several studies, there is no direct evidence of allelopathic effects on soil microbial populations. However, given the evidence of the widespread bioactive nature of chemicals derived from *S. chamaejasme*, including some with nematicidal properties [58] and activity against phytopathogenic fungi [27], direct effects of root chemicals on soil microflora might have been expected. Our findings suggest that soil chemical characteristics (organic content in particular) rather than *S. chamaejasme* factors are the stronger predictor of variation in overall fungal populations. Therefore, if root exudates from *S. chamaejasme* have any effects on fungal composition, these are probably restricted to certain components of the populations.

Given that *S. chamaejasme* sequences were detected in uninvaded soils, where this species was not recorded, the absence of established *S. chamaejasme* was likely due to soil factors rather than restricted sources of propagules. There were multiple differences in soil physiochemical parameters between sites, mostly indicative of higher soil fertility in the uninvaded site. Soil organic matter and nutrient fertility were also linked to several functional groups of fungi. However, it is likely that at least some of the soil site differences existed prior to invasion given the scale of these differences and the low above-ground annual production (1.6 tha^−1^) typical of these pastures [59]. Organic matter increases have been attributed to *S. chamaejasme* in a previous study [11], but here lower organic matter concentrations were found in the invaded soils. Using bulk density values for similar grasslands [31], differences between sites in soil organic stocks to 20 cm depth were calculated to be approximately 43 tha^−1^, equivalent to an average annual loss in excess of 2.1 tha^−1^ over the 20 years since invasion was first noted. Other differences, such as those in N and P, are likely to relate organic matter trends.

The lower C:N ratio in invaded soils is consistent with the findings of Sun et al. [11], who suggested *S. chamaejasme* may impact the quality of organic matter. The occurrence of legumes in the invaded site was considered too low to have a substantive effect on this ratio. Lower C:N ratios may also indicate higher rates of C mineralisation in invaded soils, consistent with data in Table 1 and also the findings of Sun et al. [11]. Overall, it seems likely that the invaded site was more susceptible to ingress by *S. chamaejasme* due to its lower soil fertility, leading to the indigenous plant community being less competitive, rather than *S. chamaejasme* driving such changes with consequent effects on soil fungal communities. A previous study [28] demonstrated that higher surface soil moisture (0–10 cm) favoured grass species competition with *S. chamaejasme*; this was not supported by our findings, but moisture levels may have been affected by rainfall prior to sampling.

## 5. Conclusions

The main aim of this research was to investigate potential interactions between *S. chamaejasme* and soil fungi. In terms of total fungal community composition, the evidence for any such interactions was limited. There was, however, evidence of potential influences on particular functional groups and individual species. Whether any such plant–fungal interactions contribute to invasion success is unclear, but our findings suggest the need for further research under controlled experimental conditions to explore this relationship. In addition to potential plant–fungal feedbacks, results reported here suggest that lower inherent soil fertility may be an important factor affecting the susceptibility of grassland systems to *S. chamaejasme* ingress and persistence. Soil moisture, in particular subsoil reserves [28], are also likely to alter competition between indigenous species and the deeper rooting *S. chamaejasme.* Other factors such as over-grazing, with selective herbivory and increased trampling, are known drivers of invasion.

## Figures and Tables

**Table 1:** Total soil nutrient and moisture contents (mean ± standard error, *n* = 25 per site).

	Uninvaded	Invaded	*p*-Value
% K	1.96 ± 0.016	2.02 ± 0.017	0.0487
% P	0.06 ± 0.001	0.05 ± 0.002	0.0042
% N	0.29 ± 0.043	0.22 ± 0.015	0.0009
% OM	5.72 ± 0.217	4.02 ± 0.287	0.0002
C:N ratio	11.4 ± 0.081	10.7 ± 0.089	<0.0001
% H_2_O	26.8 ± 0.816	28.2 ± 0.514	0.136
pH	7.85 ± 0.061	7.98 ± 0.082	0.176

C = carbon, N = nitrogen, P = phosphorous, K = potassium, OM = organic matter, % H_2_O = gravimetric moisture.

**Table 2:** Population diversity indices and percentage of fungi operational taxonomic units (OTUs) identified to phylum level (mean ± standard error). Significant differences (*p* < 0.05 on the basis of ANOVAs) between the uninvaded and the invaded site were indicated by different letters (*n* = 25 per site).

	Uninvaded	Invaded
**Population diversity indices**		
OTU Count	265 ± 8.38a	192 ± 7.08b
Inverse Simpson Index (OTU)	21.36 ± 1.72	17.58 ± 1.42
Shannon Index (OTU)	3.85 ± 0.06a	3.57 ± 0.08b
**% Fungi identified to phylum**		
Ascomycota	51.85 ± 1.87b	63.36 ± 2.09a
Basidiomycota	23.78 ± 1.89	19.35 ± 1.80
Zygomycota	16.8 ± 0.99a	12.8 ± 1.14b
Glomeromycota	1.44 ± 0.12a	0.49 ± 0.05b
Chytridiomycota	0.29 ± 0.05	0.22 ± 0.09
Unclassified fungi	4.06 ± 0.92	3.22 ± 0.54
Fungi unidentified	1.70 ± 0.43	0.54 ± 0.14

Significant differences (*p* < 0.05 on the basis of ANOVAs) between the uninvaded and the invaded site were indicated by different letters.

**Table 3:** SIMPER analysis for 10 species contributing most to the dissimilarity (25.16%) between the two sites (*n* = 25 each). Av.Ab: average abundance; Av.Di: average dissimilarity; Diss/SD: dissimilarity/standard deviation; Contr%: contribution to the observed dissimilarity, % of total; Cum.%: cumulative contribution %.

Species	UNITE SH ID	Uninvaded Av.Ab	Invaded Av.Ab	Av.Di	Diss/SD	Contr%	Cum.%
*Archaeorhizomyces* sp.	SH197151.06FU	7.86%	6.55%	5.14	0.93	6.52	6.52
*Mortierella* sp.	SH211066.06FU	4.28%	4.83%	2.32	1.13	2.94	9.46
*Mortierella polygonia*	SH211068.06FU	4.59%	3.24%	2.26	1.03	2.87	12.33
Sordariomycetes OTU 12		1.60%	2.33%	1.69	0.38	2.15	14.48
Mortierellales OTU 8		3.36%	0.01%	1.68	1.28	2.13	16.61
Ascomycota sp.	SH209335.06FU	1.63%	2.64%	1.42	0.75	1.80	18.41
*Humicola nigrescens*	SH234919.06FU	0.37%	2.97%	1.40	0.82	1.78	20.19
*Wallemia* sp.	SH230273.06FU	2.31%	1.14%	1.39	0.41	1.76	21.95
*Hygrocybe* sp.	SH190651.06FU	1.34%	1.50%	1.29	0.47	1.64	23.59
Agaricales OTU 20		2.47%	0.00%	1.23	0.48	1.57	25.16
